# SEED-Net: statistical echo evidence and detail modeling for ultrasound lesion classification across multiple organ-specific datasets

**DOI:** 10.3389/fmed.2026.1942733

**Published:** 2026-08-26

**Authors:** Qiong Liu, Chaofan Li

**Affiliations:** 1 School of Medical Imaging, Jiangsu Medical College, Yancheng, China; 2 Affiliated Hospital 6 of Nantong University, Yancheng Third People's Hospital, Yancheng, China

**Keywords:** computer-aided diagnosis, deep learning, feature fusion, lesion classification, ultrasound imaging

## Abstract

**Objective:**

Discriminative information in ultrasound images is often manifested as local boundary changes, internal echo heterogeneity and differences in fine-scale structure. However, it is susceptible to interference from speckle and uncorrelated background responses. This paper proposed the Statistical Echo Evidence and Detail Network (SEED-Net) for ultrasound lesion classification across multiple organ-specific datasets.

**Methods:**

SEED-Net comprises Foreground Tissue Gate, Guided Detail Unit, Channel Recalibration and Statistical Echo Evidence Head, which are used, respectively, for modulating the response of foreground tissue, modelling residuals of local structure and detail, recalibrating channel features, and fusing statistical evidence from medium and deep layers. Each prior branch uses a near-zero gate so that its initial contribution to the residual backbone is approximately zero. The model was trained on three ultrasound datasets, TN5000, SMC-LUD and PU2756, for the classification of ultrasound images of thyroid nodules, liver lesions and lung tumours, respectively. All experiments utilised whole-image input, random initialisation and independent training with five random seeds, and the model was compared with convolutional networks, visual transformers, hybrid architectures and visual state-space models.

**Results:**

SEED-Net achieved accuracy values of 0.8702 ± 0.0055, 0.9851 ± 0.0075, and 0.6054 ± 0.0086 on TN5000, SMC-LUD, and PU2756, respectively, with corresponding Macro F1-scores of 0.8384 ± 0.0048, 0.9850 ± 0.0075, and 0.5785 ± 0.0229. It achieved the highest or joint-highest Macro F1-score among the evaluated learning-based models on all three datasets, although its absolute performance on PU2756 remained limited. Ablation experiments demonstrate that the modelling of local details in the backbone and the statistical evidence classification head play complementary roles. Grad-CAM visualisations showed more compact activation patterns around lesion-related regions in the displayed examples, providing qualitative observations of the model responses.

**Conclusion:**

By integrating local detail modelling with multi-layer statistical evidence, SEED-Net achieves a favourable balance between performance and computational complexity in ultrasound classification tasks across different organs and varying levels of difficulty.

## 1 Introduction

Ultrasound, characterised by its radiation-free nature, real-time capability and accessibility, is widely used for the imaging assessment of lesions in the thyroid, liver, and lungs (1). Unlike natural image classification, discriminative information in ultrasound images is typically concentrated in fine-grained regions such as lesion boundaries, internal echoes, local hyperechoic areas and changes in adjacent tissues (2), while being subject to speckle noise, gain variations and changes in the field of view (3). Lesions of different categories exhibit similar overall grey-scale distributions, requiring models to extract robust discriminative evidence from limited local structural differences (4).

Existing convolutional networks can effectively model local textures and hierarchical structures. However, conventional convolutional features tend to respond simultaneously to both lesion details and speckle fluctuations (5). Transformers and visual state-space models enhance the ability to model global dependencies, but optimisation stability and fine-grained representation capabilities remain dependent on specific architectural designs (6). Visual state-space models utilise linear computational complexity to model long-range spatial dependencies, yet their local representation performance is equally dependent on scanning methods, convolutional embeddings and multi-scale structural design (7). Furthermore, conventional global average pooling compresses spatial features into a single mean description, making it difficult to simultaneously preserve strong local responses, overall echo levels and spatial dispersion. Extracting local details without unduly amplifying high-frequency noise, while integrating statistical information across different levels, is a key challenge in ultrasound lesion classification.

This paper proposed SEED-Net for multi-organ ultrasound lesion classification, introducing the Foreground Tissue Gate (FTG), Guided Detail Unit (GDU) and Channel Recalibration into the residual convolutional backbone. The FTG modulates foreground tissue features based on the difference between local responses and the neighbourhood background. The GDU filters out locally significant responses through local structure estimation and detail residual reconstruction, and Channel Recalibration is used to supplement cross-channel feature interactions. During the classification stage, the Statistical Echo Evidence Head (SEEH) jointly extracts GeM, mean and standard deviation descriptions, and fuses mid-level and deep-level features to form a more comprehensive representation of echo evidence. Each prior branch employs near-zero gating, enabling the network to maintain a stable residual baseline during the early stages of training, before gradually learning the corresponding modulation intensities based on supervisory signals.

## 2 Related works

### 2.1 Deep architectures for ultrasound lesion classification

Convolutional neural networks are the most widely used architecture for the classification of ultrasound lesions. Han (8) validated the effectiveness of GoogLeNet, combined with histogram equalisation, cropping and edge enhancement, for the classification of benign and malignant breast ultrasound findings, thereby achieving synergistic effects in computer-aided diagnosis. Michal Byra (9) improved the breast lump classification task based on transfer learning and colour transformation in the matching layer. Zhu (10) trained separate thyroid and breast models based on deep convolutional neural network architectures, outperforming radiologists in the classification of both types of cancer. Kim (11) compared the performance of three deep learning models, VGG16, VGG19 and ResNet, in the discrimination of thyroid ultrasound lesions. ResNet improves the optimisation stability of deep networks through residual connections, while DenseNet utilises dense connections to facilitate multi-layer feature reuse, both architectures are widely applied in the benign-malignant differentiation of breast and thyroid ultrasound lesions (12, 13). EfficientNet maintains convolutional inductive bias while improving parameter efficiency by jointly scaling network depth, width and input resolution, and has gradually become an efficient backbone in ultrasound classification research. Yu (14) extracted statistical features based on the TI-RADS system, after dimensionality reduction via PCA, these were fused with the feature maps of EfficientNet B3 for the classification of thyroid ultrasound images. Latha (15) addressing the difficulties traditional CNNs face in handling class imbalance and subtle textural variations in breast ultrasound image classification, employed EfficientNet-B7 in combination with data augmentation, an early stopping strategy and Grad-CAM explainability techniques. This approach achieved a classification accuracy of 99.14% on the BUSI dataset, providing reliable automated assistance for the early diagnosis of breast cancer. CNNs are capable of reliably extracting local textures and hierarchical structures, through transfer learning, input augmentation or model ensembling, they can enhance their ability to characterise lesion textures, edges and morphological features. Recent studies in other medical imaging domains have also combined modality-specific image enhancement or handcrafted descriptors with multi-branch CNN feature fusion for image classification (16, 17). However, their results are typically influenced by data scale, pre-training methods and the range of lesion inputs (18).

With the development of self-attention mechanisms, Vision Transformers and their hybrid architectures have begun to be applied to ultrasound image analysis. Zhou (19) explored the application of ViT, ensemble learning and knowledge distillation in the classification of benign versus malignant breast ultrasound images, finding that the ensemble model outperformed a single ViT in terms of classification performance. Saad (20) proposed a BreastUS Transformer model incorporating self-attention mechanisms for the automatic classification of breast ultrasound images, achieving a performance improvement of at least 4% compared to state-of-the-art CNNs. Recognising that patch may compromise local morphological information, Huseyin (21) utilised DenseNet121 backbone, combined with spatial compression incentives and Swin Transformer, to capture spatial details and long-range contextual features for the classification of breast tumour ultrasound images. Wang (22) proposed a multi-stage fusion network combining CNN and Transformer, which integrates features through four-stage interaction modules and introduces a convolutional block attention mechanism, achieving excellent classification performance in both internal and external validation. Huang (23) proposed the Swin-Residual Transformer model, incorporating residual blocks and triplet loss into the Swin Transformer to enhance sensitivity to both global and local features of thyroid nodules. Atutxa (24) combined EfficientNet-B7 with the Swin Transformer for the classification of ovarian tumour ultrasound images, advancing the ovarian ultrasound classification task from a focus on mere accuracy to an explainable clinical decision support framework that accounts for uncertainty. Zhao (25) utilised pre-training to extract local multi-scale features, combined with a lightweight Transformer for global information interaction, for the diagnosis of focal liver lesions in contrast-enhanced ultrasound images.

Visual state space models have emerged as the latest backbone paradigm in the field of ultrasound classification. Nasiri-Sarvi (26) compared Mamba-based models with CNNs and ViTs on ultrasound datasets, the Mamba models performed statistically significantly better than non-Mamba models and were able to effectively capture long-range dependencies while maintaining inductive bias. Laicu-Hausberger (27) utilised SSM units to model large-scale spatial dependencies for lesion classification in breast ultrasound. Rao (28) combined a pyramid structure for multi-scale feature extraction, the Mamba model for modelling long-range dependencies, and a serial-channel spatial attention module to enhance feature representation, achieving excellent results on thyroid ultrasound images and validating the potential of hybrid modelling combining Mamba with other paradigms. Aniq (29) utilised Mamba for pre-processing breast ultrasound images and k-fold cross-validation, achieving performance comparable to CNN/Transformer baselines while incurring lower computational costs. He (30) utilised MambaCA-Net to extract static and dynamic breast ultrasound features from single-frame image streams and continuous-frame video streams respectively, achieving a marked improvement in classification performance, its lightweight architecture and robust feature extraction capabilities hold significant potential for breast cancer detection.

Overall, CNNs, transformers, hybrid networks and visual state-space models provide local representation, global interaction and efficient long-range modelling capabilities, respectively. These architectures primarily enhance performance by expanding the receptive field or altering the manner in which features interact, they do not directly distinguish between tissue structure, lesion details and speckle fluctuations in ultrasound images. For ultrasound lesion tasks with limited sample sizes, complex global structures have also failed to consistently achieve fine-grained representations superior to those of convolutional models. In addition to general-purpose network architectures, it is necessary to establish more targeted feature modelling approaches that take into account the characteristics of ultrasound imaging.

### 2.2 Ultrasound-specific representation and evidence modeling

To address issues such as blurred lesion boundaries, similar local echoes and complex background tissue in ultrasound imaging, some studies have enhanced classification features through lesion localisation, segmentation or clinical *a priori* information. Ryu (31) developed a user-click-based convolutional neural network system that combines segmentation and classification for the ultrasound diagnosis of four types of liver lesions: liver cysts, haemangiomas, metastases and hepatocellular carcinoma. Luo (32) first trained a segmentation network to generate tumour-segmented enhanced images, then utilised two parallel networks to extract features from the original and enhanced images respectively, thereby guiding the image classification task. Similarly, Bryar Shareef (33) proposed the Hybrid-MT-ESTAN hybrid multi-task deep neural network, combining CNN and Swin Transformer components for tumour classification and segmentation in breast ultrasound images, effectively addressing the shortcomings of CNNs in global modelling and the local pattern distortion in ViT. Zhang (34) utilised an improved U-Net++ for nodule segmentation, followed by a multi-task convolutional neural network to classify four types of malignant features and calculate risk grades based on the TI-RADS guidelines, thereby providing reports more closely aligned with clinical practice. Bobowicz (35) compared multi-class segmentation methods for breast ultrasound images and proposed a classification method integrating bounding boxes, mask classifiers and voting strategies, this combined segmentation–classification approach demonstrated superior performance in BI-RADS multi-class classification. Such methods can explicitly delineate lesion regions, enabling the model to learn morphological and echogenicity features relevant to clinical diagnosis. However, they often rely on user interaction, lesion contour annotation, ROI information or additional diagnostic attributes.

Some studies have also focused directly on modelling the morphology, edges and local texture of ultrasound lesions. Zhong (36) established a multi-scale fusion framework for breast ultrasound lesions, generating multi-scale representations through spatial context aggregation, employing a fusion strategy to capture morphological features, and introducing an isotropic feature module to enhance texture modelling. Cui (37) adaptively extracted and fused features from within tumours, around tumours, and in transitional regions within breast ultrasound images using an enhanced joint tumour module and a branch fusion module. Explicit modelling of lesion edges is also a key aspect of ultrasound lesion features. Xu (38) developed a fine-grained BI-RADS classification system that integrates tumour edge information, utilising jointly trained edge pseudo-labels and a classification network to highlight edge regions. Vakanski (39) utilised visual saliency priors to guide the network’s attention towards breast lesion regions, thereby reducing interference from irrelevant locations during feature learning. With regard to frequency pattern and boundary texture modelling, Rhyou (40) proposed HCC-Net, based on the discrete wavelet transform, which enhances the recognition of hepatocellular carcinoma through coarse-to-fine hierarchical lesion detection and a pattern-enhanced classifier. Saini (41) utilised two-dimensional variational modal decomposition to extract boundary and texture patterns associated with breast lesions, combining hybrid pooling and attention mechanisms to achieve classification without prior segmentation. Research focusing on multi-scale morphology and texture, lesion edges and surrounding regions, saliency-guided approaches, and frequency decomposition has revealed diverse modelling approaches for spatial structure and frequency features. This demonstrates that lesion boundaries, adjacent tissue, and different frequency components all contain effective discriminative cues, some approaches still rely on explicit localisation, pre-segmentation, or additionally generated lesion images.

Feature aggregation methods also influence classification representations. Conventional global average pooling retains only the average response for each channel, making it difficult to simultaneously capture both strong local activations and spatial variations. Generalised Mean Pooling modulates between average pooling and max pooling via a learnable exponent, while global covariance pooling enhances the descriptive power of feature distributions through second-order statistics (42). Existing ultrasound classification methods focus primarily on ROI extraction, attention enhancement or multi-scale spatial fusion, while lacking a unified model for the salience of local details relative to the neighbourhood background, as well as the diverse statistical evidence present in deep features. With this in mind, this paper jointly models foreground tissue responses, local structures and detail residuals on complete ultrasound images, and forms evidence for lesion classification through multi-statistic pooling and the fusion of features from the middle and deep layers. Unlike methods that rely on lesion masks, edge pseudo-labels, explicit frequency decomposition, or separately generated lesion images, SEED-Net does not introduce additional annotations or fixed signal decomposition. Its individual operators are conventional, but they are organised according to three different stages of evidence construction: early spatial tissue-response calibration, intra-backbone structure–detail modulation, and cross-level statistical evidence aggregation. The proposed contribution therefore lies in the hierarchical integration of these functions rather than in claiming novelty for each elementary operator.

## 3 Methods

### 3.1 Overall design

**Figure 1:**
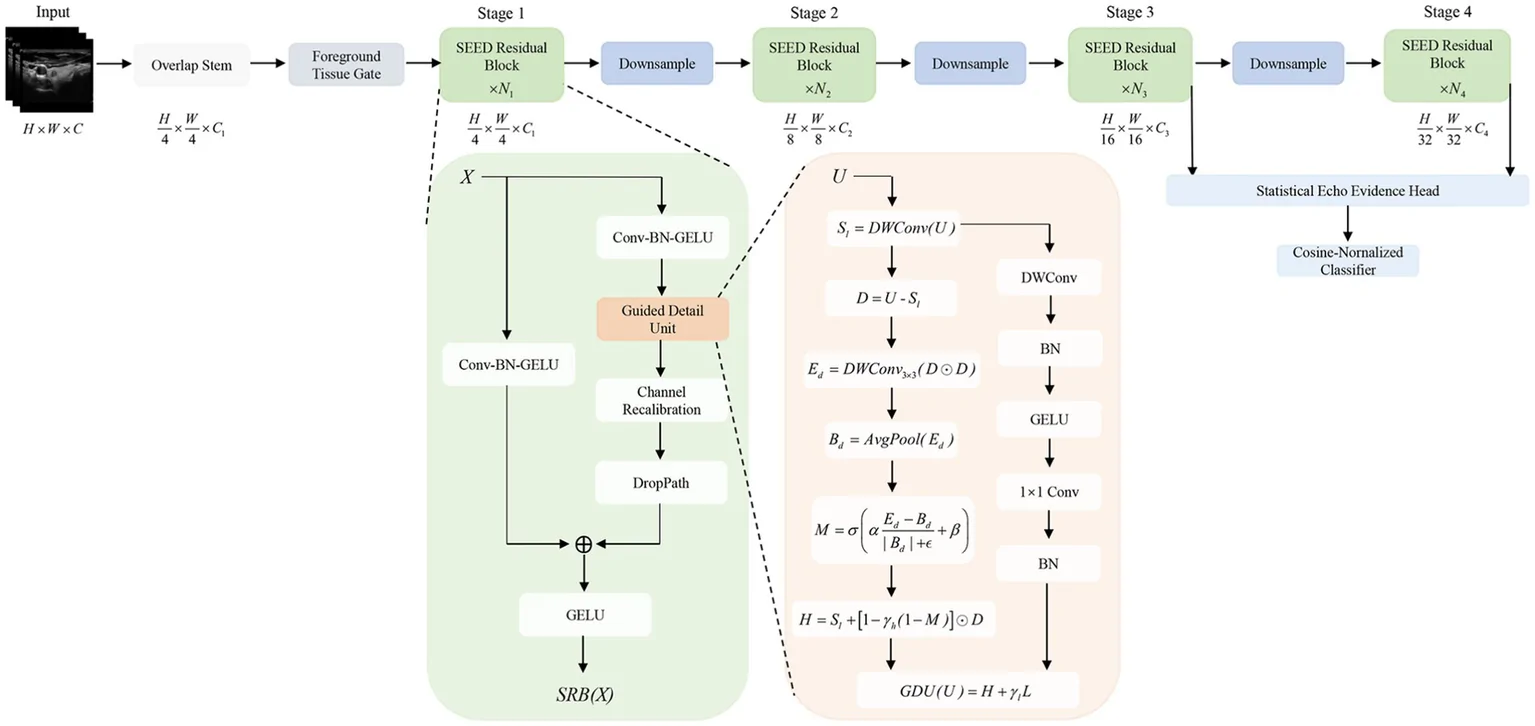
Overall architecture and details of SEED-Net.

SEED-Net is organised as a hierarchical evidence-modelling framework for ultrasound image classification. FTG is positioned after the stem and calibrates early spatial responses according to their relative difference from the local neighbourhood. GDU is embedded within the residual backbone and models a learnable local structural estimate together with its detail residual, allowing locally prominent residual responses to be modulated under classification supervision. SEEH operates after feature extraction and aggregates mid-level and deep-level evidence through GeM, mean, and standard deviation statistics. Thus, the three components act on different representation levels: FTG performs early spatial response calibration, GDU performs intra-backbone structure–detail modulation, and SEEH performs cross-level statistical evidence aggregation. Their common principle is relative evidence modelling, but their inputs, positions, and functions are different. The overall logic of SEED-Net is as Equations (1)–(28):


$$y=\text{SEEH}(\mathrm{SR}B_{1-4}(\mathrm{FTG}(\text{Stem}(X))))$$ (1)

where *FTG()* denotes the Foreground Tissue Gate, features sequentially pass through four stages comprising SEED residual blocks, and *SEEH()* denotes the Statistical Echo Evidence Head. The overall architecture of SEED-Net is shown in Figure 1.

### 3.2 Datasets and preprocessing

This study collated and evaluated three 2-dimensional ultrasound image datasets. TN5000 (43) is a dataset for the binary classification of thyroid nodules as benign or malignant, comprising a total of 5,000 images categorised as benign or malignant. It follows the official train/validation/test split, with 3,500 images in the training set, 500 in the validation set and 1,000 in the test set. SMC-LUD (44) is a two-class classification dataset for ultrasound lesions of the liver, designed to distinguish between hepatocellular carcinoma (HCC) and haemangioma. This study utilised only the clean version of the dataset, from which caliper markers had been removed, comprising a total of 2,656 images, with 1,858, 530 and 268 images allocated to the training, validation and test sets, respectively. PU2756 (45) is a pulmonary tumour ultrasound dataset comprising 2,756 images for benign–malignant classification. The adopted split contained 1,763 training images, 441 validation images, and 552 test images. The test set was class-imbalanced, with malignant cases accounting for approximately 63% of the samples.

According to the original dataset descriptions, the benign–malignant labels in TN5000 and PU2756 were established using biopsy or pathological findings. In SMC-LUD, HCC cases were histopathologically confirmed through surgical resection or biopsy, whereas haemangioma cases were established from characteristic radiological findings. Therefore, although the three datasets provide clinically established class labels, their reference-standard procedures are not identical, and the results are interpreted within each dataset-specific classification setting.

The class distributions were not identical across the three datasets. For TN5000, the benign/malignant sample counts were 1032/2468 in the training set, 125/375 in the validation set, and 269/731 in the test set. For SMC-LUD, the HCC/haemangioma counts were 972/886, 277/253, and 140/128, respectively. For PU2756, the benign/malignant counts were 640/1123, 160/281, and 201/351, respectively. SMC-LUD was therefore approximately balanced, whereas TN5000 and PU2756 contained a larger proportion of malignant samples.

All datasets have been uniformly organised into an ImageFolder structure. For TN-5000, the official split is used, and the bounding boxes in the XML are not utilised for lesion cropping. For SMC-LUD, only the clean version is used, neither original_data nor split_for_train is utilised. For PU2756, only the images folder and five_fold_split.xlsx are used, the masks folder is not utilised. All images were EXIF-corrected and converted to RGB format, followed by deterministic offline preprocessing. Obvious outer borders and ultrasound device-interface regions were removed to reduce non-anatomical layout cues. The whole-image input therefore refers to the complete processed ultrasound imaging region, including the lesion and adjacent tissues, rather than the original device screenshot. The images were resized with preserved aspect ratio and padded to 224 × 224 pixels before being saved as PNG files. No lesion-centred ROI, bounding-box, mask, or localisation crop was used. During the training phase, only mild geometric and greyscale intensity enhancement was applied to the training set, no enhancement was applied to the validation or test sets. TN5000, SMC-LUD, and PU2756 were trained and evaluated independently rather than pooled into a single classification task. Consequently, acquisition differences across datasets were not directly available to one classifier as cross-dataset class cues.

### 3.3 Foreground tissue gate

Ultrasound images often contain background regions, local shadows, gain variations and low-contrast tissue boundaries. To enhance local tissue response, SEED-Net introduces foreground tissue gating after the stem stage. Given the input features $X_{s}\in {\mathbb{R}}^{N\times C\times H\times W}$ , the average absolute response across channels is first calculated:


$$E_{f}=\frac{1}{C}\sum_{c=1}^{C}|X_{s,c}|,$$

where $E_{f}$ denotes the spatial response map, and *c* is the channel index, average pooling is used to estimate the local background baseline:


(3) $$B_{f}=\text{AvgPool}(E_{f}),$$

where $B_{f}$ denotes the average response of the local neighbourhood. Based on the relative difference between the response at the current position and the local baseline, a foreground gating map is constructed:


(4) $$G_{f}=\sigma (\frac{E_{f}-B_{f}}{|B_{f}|+\in }),$$

where $G_{f}$ denotes the initial gating map. To avoid global amplification caused by overall brightness shifts, spatial de-meanning is further applied:


(5) $$\tilde{G_{f}}=G_{f}-\frac{1}{\mathrm{HW}}\sum_{h=1}^{H}\sum_{w=1}^{W}G_{f,:,:,h,w}$$ (2)

The final output is:


$$\mathrm{FTG}(X_{s})=X_{s}⊙(1+\gamma_{f}\tilde{G_{f}}),$$

where $\gamma_{f}$ is a learnable scalar initialised to 0, which does not disrupt the distribution of backbone features in the early stages of training, but instead gradually learns the intensity of foreground organisation modulation with the support of the supervisory signal.

### 3.4 SEED residual block

The SEED residual block is the basic unit of the backbone network, the main branch first passes through two 3 × 3 convolutional units, followed sequentially by GDU module and channel recalibration:


(7) $$\mathrm{SRB}(X_{b})=\phi (T(X_{b})+\text{DropPath}(\mathrm{ECA}(\mathrm{GDU}(\text{Conv}(X_{b})))))$$ (6)

#### 3.4.1 Guided detail unit

The Guided Detail Unit (GDU) is designed to modulate structure-detail representations within the residual backbone. GDU does not assume that all high-frequency responses are diagnostically relevant, because both lesion-related details and speckle fluctuations may appear as high-frequency structures. Instead, it first obtains a learnable local structural estimate and defines the remaining response as a detail residual. The local second-order energy of this residual is then compared with its neighbourhood baseline to construct a relative saliency mask. Given an input feature *U*, the local structural estimate is obtained by depthwise convolution:


$$S_{l}=\text{DWConv}(U),$$

where $S_{l}$ denotes the learnable local structural estimate, and the corresponding detail residual is defined as:


(9) $$D=U-S_{l}$$ (8)

Subsequently, a learnable deep convolution is applied to the element-wise squares of the detail residuals to obtain the local second-order detail response:


$$E_{d}=\text{DWCon}v_{3\times 3}(D⊙D),$$

where $E_{d}$ represents the intensity of the local detail response. A neighbourhood reference is then obtained via average pooling:


(11) $$B_{d}=\text{AvgPool}(E_{d})$$ (10)

A saliency mask is constructed based on the difference between the response at the current position and the local baseline:


$$M=\sigma (\alpha \frac{E_{d}-B_{d}}{|B_{d}|+\in }+\beta )$$ (12)

α and β are learnable scalars, initialised to 1 and 0, respectively. The closer *M* is to 1, the more prominent the detail response at that position is relative to the local background. The closer *M* is to 0, the closer the detail at that position is to local background fluctuations. This operation does not explicitly label a residual response as pathological detail or speckle. Instead, it measures whether the response is relatively prominent with respect to its local context. Residual responses that are similar to neighbourhood fluctuations tend to receive weaker modulation, whereas relatively salient responses can be retained. Their diagnostic relevance is subsequently determined by the end-to-end classification objective. GDU should therefore be interpreted as a supervised relative-detail modulation mechanism rather than a deterministic speckle-removal module.

During the reconstruction phase, GDU uses the saliency mask to gate the detail residuals:


$$H=S_{l}+[1-\gamma_{h}(1-M)]⊙D,$$

where *H* denotes the reconstructed features modulated by details, and $\gamma_{h}$ is a learnable scalar used to control the suppression intensity in non-salient detail regions. When $\gamma_{h}=0$ , this branch maintains an identity mapping.

Concurrently, the low-frequency structural components undergo deep separable convolutions for structural refinement:


(14) $$L=\mathrm{BN}(\mathrm{Con}v_{1\times 1}(\phi [\mathrm{BN}(\text{DWConv}(S_{l}))]))$$ (13)

The final output of GDU is:


$$\mathrm{GDU}(U)=H+\gamma_{l}L$$ (15)

Consequently, GDU degenerates to an identity mapping during the initial training phase, allowing the model to begin from a stable residual convolutional backbone before gradually learning the required degree of detail modulation and local structural refinement.

#### 3.4.2 Channel recalibration

The lightweight channel re-calibration module addresses cross-channel dependencies. The input feature *V* first undergoes global average pooling to obtain channel descriptors:


$$z=\mathrm{GAP}(V)\in {\mathbb{R}}^{N\times C}$$ (16)

Subsequently, one-dimensional convolution is used to model local channel interactions along the channel dimension:


a=σ(Conv1D(z)) (17)

The final output is:


$$\mathrm{ECA}(V)=V⊙[1+\gamma_{e}(a-0.5)],$$

where $\gamma_{e}$ is a learnable scalar, channel modulation is centred around 0.5, this initialisation prevents channel attention from causing excessive perturbation to the features during the early stages of training.

### 3.5 Statistical echo evidence head

SEED-Net uses statistical echo evidence headers to fuse mid-level and deep-level features, defining generalised mean pooling for the input feature *F* as follows:


(19) $$g(F)={\left[\mathrm{GAP}({\left(\text{ReLU}(F)+\epsilon \right)}^{p})\right]}^{1/p},$$

where $g(F)\in {\mathbb{R}}^{N\times C}$ represents the GeM features, and *p* is a learnable scalar initialised to 3. *ReLU()* ensures that the pooling denominator remains non-negative. The global mean and spatial standard deviation are defined as follows:


(20) μ(F)=GAP(F) (18)

$$s(F)=\mathrm{St}d_{h,w}(F)$$ (21)

For deep-layer features $F_{d}$ , the GeM features serve as the primary representation, with mean and standard deviation statistics introduced via near-zero gating:


$$f_{d}=g(F_{d})+\gamma_{s}W_{s}[\mu (F_{d});s(F_{d})],$$

where $f_{d}$ represents the deep-layer statistically enhanced features, $W_{s}$ is a linear projection, and $\gamma_{s}$ is a learnable scalar.

For mid-layer features $F_{m}$ , GeM, mean and standard deviation are extracted simultaneously:


(23) $$f_{m}=W_{m}[g(F_{m});\mu (F_{m});s(F_{m})]$$ (22)

The final fused feature is:


$$f=f_{d}+\rho f_{m}$$ (24)

ρ is a learnable scalar, during the initialisation phase, the model relies solely on the deep primary features, while during training it automatically determines the extent to which mid-layer echo evidence is incorporated based on the data. Dropout is applied to the fused features:


$$\bar{f}=\text{Dropout}(f)$$ (25)

Prior to classification, the features and class weights are normalised as follows:


$$\hat{f}=\frac{\hat{f}}{{\hat{f}}_{2}},{\hat{w}}_{k}=\frac{w_{k}}{w_{k2}},$$

where $\hat{f}$ is the normalised feature and ${\hat{w}}_{k}$ is the normalised weight, the final logits are:


(27) $$y_{k}=\tau {\hat{f}}^{⊤}{\hat{w}}_{k},$$

where $y_{k}$ denotes the logit for class *k*, and τ is a learnable temperature parameter. The cosine-normalised classifier constrains classification decisions to angular space, which helps mitigate the impact of differences in class sample sizes on the norm of the classifier’s weights.

### 3.6 Near-zero initialisation strategy

All ultrasound-related priors in SEED-Net employ near-zero gating, primarily comprising foreground tissue gating intensity, detail residual modulation, low-frequency structure refinement, channel re-calibration intensity, deep statistical enhancement, and mid-layer evidence fusion. Consequently, the added modulation branches contribute approximately zero at initialization, and the network begins from the corresponding residual backbone mapping. During training, the gate parameters are jointly optimized with the remaining network parameters, allowing the contribution of each branch to be learned from the classification objective. Near-zero initialization is therefore used as an initialization condition rather than being treated as an independently validated performance-enhancing component.

### 3.7 Loss function

Training is carried out using a cross-entropy loss with class weights, and label smoothing is applied to reduce the model’s overfitting to hard labels. Let the model’s predicted probability for class *k* is $p_{k}$ , the smoothed label is ${\tilde{q}}_{k}$ , and the class weights is $w_{k}$ , the loss function is:


(28) $$ℒ=-\sum_{k=1}^{K}w_{k}{\tilde{q}}_{k}\log p_{k}$$ (26)

The class weights were used to mitigate class imbalance in the training set, while the label smoothing coefficient was set to 0.05 to improve training stability.

### 3.8 Implementation details

All experiments used 224 × 224 images as input, and no externally pre-trained weights were used. The comparison models retained their architecture-specific default configurations where applicable, rather than sharing one identical hyperparameter setting. The dataset partitions, input resolution, preprocessing procedure, model-selection criterion, evaluation metrics, and five-run evaluation protocol were kept consistent across models. Image standardisation was calculated solely from the training set of each dataset and was applied to the corresponding training, validation, and test sets. The training set underwent random horizontal flipping, mild affine transformations, adjustments to brightness and contrast, Gaussian noise, and Gaussian blurring, whereas the validation and test sets underwent only tensor transformation and standardisation. For SEED-Net, AdamW was used with a batch size of 32, an initial learning rate of 2e-4, and a weight decay of 1e-4. Linear warm-up was applied for the first five epochs, followed by a cosine-annealing strategy with a minimum learning rate of 1e-6. The best model was selected based on the macro-average F1 score on the validation set, with patience of 25 and maximum of 100 training epochs. Each experiment was independently repeated using five predefined random seeds, and the mean and standard deviation of the five test results were reported to quantify variability arising from random initialization and stochastic optimization. These mean ± SD values describe run-to-run variability and should not be interpreted as confidence intervals for clinical performance. The repeated runs were interpreted descriptively, and no claim of statistical significance was made from the seed-level results. Evaluation metrics included Accuracy, Macro Precision, Macro Recall, and Macro F1-score. Macro Recall was calculated as the unweighted mean of the class-wise recalls, because all tasks in this study were binary, Macro Recall was equivalent to balanced accuracy.

## 4 Results

This section evaluates SEED-Net in terms of its overall classification performance, stability across different ultrasound tasks, and the actual contributions of each core module. Firstly, the proposed model is compared with representative convolutional networks, visual Transformers, hybrid networks and visual state-space models. It is trained and tested independently on three classification datasets, TN5000, SMC-LUD, and PU2756 to analyse the performance of the proposed model in ultrasound classification tasks for thyroid nodules, liver lesions and lung tumours. The comparative results represent performance under random initialization with the corresponding architecture-specific configurations and should not be interpreted as the maximum performance attainable through external pre-training or extensive model-specific hyperparameter optimization.

### 4.1 Thyroid nodule classification results on TN5000

The TN-5000 is used for the binary classification of thyroid nodules as benign or malignant. The main challenge in this task lies in the fact that the overall grey-scale distributions of benign and malignant nodules may be quite similar, classification often relies on fine-grained features such as border morphology, internal echogenic heterogeneity and local hyperechoic areas. Table 1 presents the results of the overall classification performance evaluation for each model.

**Table 1:** Comparative classification performance on the TN5000 dataset.

Structure	Model	Accuracy	Macro precision	Macro recall	Macro F1-score	Params	FLOPs
CNN-style	ResNet50	0.8232 ± 0.0164	0.7755 ± 0.0193	0.7762 ± 0.0341	0.7748 ± 0.0274	23.5121	4.1317
	EfficientNet-V2	0.8656 ± 0.0108	0.8297 ± 0.0141	0.8301 ± 0.0176	0.8293 ± 0.0139	20.1826	2.9007
	ConvNeXt-V2	0.7834 ± 0.0187	0.7286 ± 0.0196	0.7370 ± 0.0202	0.7307 ± 0.0180	27.8003	4.4548
	RepViT	0.7758 ± 0.0181	0.7164 ± 0.0214	0.7202 ± 0.0189	0.7179 ± 0.0201	13.6184	2.3400
Transformer-style	Vision-Transformer	0.6598 ± 0.0449	0.5915 ± 0.0396	0.6005 ± 0.0475	0.5897 ± 0.0445	85.6097	16.8477
	Swin-Transformer-V2	0.7510 ± 0.0056	0.6842 ± 0.0102	0.6861 ± 0.0205	0.6845 ± 0.0156	27.5659	4.5161
	EfficientViT	0.7394 ± 0.0268	0.6712 ± 0.0326	0.6749 ± 0.0337	0.6725 ± 0.0329	3.9620	0.2031
Hybrid-style	CoAtNet	0.8490 ± 0.0154	0.8075 ± 0.0198	0.8241 ± 0.0103	0.8139 ± 0.0142	16.9913	3.3509
	MaxViT	0.8410 ± 0.0181	0.8007 ± 0.0251	0.8165 ± 0.0125	0.8048 ± 0.0134	28.4540	4.8908
	MedViT	0.8640 ± 0.0118	0.8265 ± 0.0164	0.8353 ± 0.0143	0.8298 ± 0.0122	31.1405	5.9286
Mamba-style	EfficientViM	0.7962 ± 0.0171	0.7422 ± 0.0215	0.7389 ± 0.0151	0.7399 ± 0.0171	15.0193	0.6484
	MambaVision	0.8430 ± 0.0100	0.8005 ± 0.0127	0.8292 ± 0.0115	0.8106 ± 0.0070	31.1353	4.4712
	MedMamba	0.8262 ± 0.0149	0.7790 ± 0.0189	0.7904 ± 0.0138	0.7839 ± 0.0159	13.2591	2.0244
Proposed	Seed-Net	0.8702 ± 0.0055	0.8324 ± 0.0081	0.8459 ± 0.0037	0.8384 ± 0.0048	13.0145	1.9636

On the TN5000 dataset, SEED-Net achieved the highest mean values across all four classification metrics, with Accuracy, Macro Precision, Macro Recall and Macro F1-score reaching 0.8702 ± 0.0055, 0.8324 ± 0.0081, 0.8459 ± 0.0037 and 0.8384 ± 0.0048, respectively. Compared with the second-best method, Accuracy and Macro Precision improved by 0.46 and 0.27 percentage, respectively, over EfficientNet-V2, while Macro Recall and Macro F1-score improved by 1.06 and 0.86 percentage, respectively, over MedViT.

Under the current random-initialization setting, substantial performance differences were observed among the individual comparison models. Among CNN-based models, EfficientNet-V2 performed best, with Accuracy and Macro F1-score of 0.8656 and 0.8293 respectively, significantly outperforming ResNet50, ConvNeXt-V2 and RepViT. Neither an increase in network scale nor architectural updates resulted in improved performance. Pure Transformer models performed relatively poorly overall, Vision Transformer, in particular, achieved Accuracy and Macro F1-score of only 0.6598 and 0.5897, respectively, with standard deviations of 0.0449 and 0.0445, indicating significant performance fluctuations under the current random initialisation training conditions. Swin Transformer-V2 improves stability through local window modelling, but its Macro F1-score remains at 0.6845, far below that of robust CNNs and hybrid architectures. EfficientViT has the lowest number of parameters and FLOPs, but its corresponding Macro F1-score is only 0.6725, excessive compression of model complexity limits its ability to represent fine-grained echogenic features of thyroid nodules. Among the models evaluated under the current setting, MedViT achieved an Accuracy of 0.8640 and a Macro F1-score of 0.8298, making it the comparison method whose performance most closely matched that of SEED-Net. CoAtNet and MaxViT achieved Accuracies of 0.8490 and 0.8410 respectively, but their Macro F1-scores were only moderate. Among Mamba-class models, MambaVision performed best, with Accuracy and Macro Recall of 0.8430 and 0.8292 respectively, while EfficientViM and MedMamba achieved Macro F1-scores of 0.7399 and 0.7839 respectively, the benefits of state-space modelling for this task still depend on the specific architecture and the method of local feature modelling.

In addition to its average performance, SEED-Net exhibited the lowest standard deviation among all methods across all four metrics in five independent experiments, indicating that the model demonstrates good stability against random initialisation and data variations. In terms of computational complexity, SEED-Net comprises only 13.01 M parameters and 1.96G FLOPs, representing reductions of approximately 35.5 and 32.3%, respectively, compared to EfficientNet-V2, and reductions of approximately 58.2 and 66.9%, respectively, compared to MedViT. Although EfficientViT and EfficientViM have lower computational costs, their classification performance suffers significantly. Taking into account classification accuracy, class balance, stability across repeated experiments and computational complexity, SEED-Net achieves a superior performance-complexity balance on the TN5000 dataset. SEED-Net contained 13.01 M parameters and required 1.96 G FLOPs, with serialized model size of 49.82 MB. Under the tested hardware environment, it achieved an average single-image inference latency of 4.31 ms, corresponding to a throughput of 232 FPS. These results suggest that SEED-Net has the computational potential for deployment in clinical ultrasound workstations, although its actual deployment suitability requires device-specific optimisation and prospective clinical validation.

### 4.2 Liver lesion classification results on SMC-LUD

SMC-LUD is used to distinguish hepatocellular carcinoma from hepatic haemangioma, as these two types of lesions may exhibit similar echo intensities and border characteristics in some ultrasound images. The model must utilise the internal echoes of the lesion, its relationship with surrounding tissues, and local structural differences to make this distinction. The ability of each model to learn the imaging features of the lesions themselves was examined, and the evaluation results are shown in Table 2.

**Table 2:** Comparative classification performance on the SMC-LUD dataset.

Structure	Model	Accuracy	Macro precision	Macro recall	Macro F1-score
CNN-style	ResNet50	0.9769 ± 0.0072	0.9771 ± 0.0075	0.9766 ± 0.0070	0.9768 ± 0.0072
	EfficientNet-V2	0.9821 ± 0.0081	0.9821 ± 0.0083	0.9821 ± 0.0079	0.9821 ± 0.0081
	ConvNeXt-V2	0.9701 ± 0.0053	0.9701 ± 0.0052	0.9704 ± 0.0051	0.9701 ± 0.0053
	RepViT	0.9799 ± 0.0057	0.9799 ± 0.0056	0.9797 ± 0.0057	0.9798 ± 0.0057
Transformer-style	Vision-Transformer	0.9612 ± 0.0077	0.9610 ± 0.0079	0.9615 ± 0.0075	0.9611 ± 0.0077
	Swin-Transformer-V2	0.9769 ± 0.0031	0.9770 ± 0.0031	0.9768 ± 0.0031	0.9768 ± 0.0031
	EfficientViT	0.9806 ± 0.0089	0.9807 ± 0.0089	0.9805 ± 0.0089	0.9806 ± 0.0089
Hybrid-style	CoAtNet	0.9851 ± 0.0059	0.9852 ± 0.0056	0.9850 ± 0.0061	0.9850 ± 0.0059
	MaxViT	0.9843 ± 0.0049	0.9845 ± 0.0047	0.9842 ± 0.0050	0.9843 ± 0.0049
	MedViT	0.9828 ± 0.0020	0.9827 ± 0.0020	0.9829 ± 0.0021	0.9828 ± 0.0020
Mamba-style	EfficientViM	0.9746 ± 0.0055	0.9746 ± 0.0055	0.9747 ± 0.0055	0.9746 ± 0.0055
	MambaVision	0.9791 ± 0.0043	0.9795 ± 0.0041	0.9788 ± 0.0044	0.9790 ± 0.0043
	MedMamba	0.9784 ± 0.0072	0.9784 ± 0.0073	0.9785 ± 0.0069	0.9783 ± 0.0072
Proposed	Seed-Net	0.9851 ± 0.0075	0.9850 ± 0.0074	0.9851 ± 0.0076	0.9850 ± 0.0075

The overall performance of the various models on the SMC-LUD dataset was consistently high, with accuracy ranging from 0.9612 to 0.9851 and Macro F1-score ranging from 0.9611 to 0.9850, the differences between models were significantly smaller than those observed on TN5000. As this dataset utilises a clean version with caliper labels removed, and given that the class distributions of HCC and haemangioma are relatively similar, the evaluation metrics remain highly consistent across most models. The classification results for both classes are well-balanced across the various methods, with no obvious class bias observed.

SEED-Net achieved an accuracy of 0.9851 ± 0.0075, tying with CoAtNet for the highest value, its macro recall reached 0.9851 ± 0.0076, the highest among the compared models, and its macro F1-score was 0.9850 ± 0.0075, also at the highest level. CoAtNet’s Macro Precision was slightly higher at 0.9852 ± 0.0056, while its Macro Recall and Macro F1-score were both 0.9850. SEED-Net and CoAtNet demonstrated broadly comparable average performance on this dataset. MaxViT and MedViT also achieved high results, with Macro F1-scores of 0.9843 and 0.9828 respectively, hybrid architectures generally performed quite consistently on this task. The differences between model types were primarily evident in their lower performance bounds rather than their optimal results. Among CNN models, EfficientNet-V2 achieved a Macro F1-score of 0.9821, outperforming ResNet50, ConvNeXt-V2 and RepViT. Among Transformer models, EfficientViT reached 0.9806, exceeding both Vision Transformer and Swin Transformer. The Macro F1-scores of Mamba-class models ranged between 0.9746 and 0.9790, which were generally lower than those of the best-performing hybrid architectures and SEED-Net, though the gap did not widen significantly. It can thus be seen that, in the SMC-LUD task, local modelling, hierarchical feature extraction and hybrid architectures are all capable of achieving high classification performance, with no single architecture type establishing an absolute advantage. SEED-Net achieved the highest or joint-highest average results, reaching the highest performance level in the current experiments, and is capable of effectively adapting to the ultrasound classification tasks for hepatocellular carcinoma and hepatic haemangioma.

### 4.3 Pulmonary tumor classification results on PU2756

PU2756 is used for the classification of benign and malignant lung tumours. This task is affected by the overlap in the appearances of benign and malignant lesions, as well as variations in the field of view, placing higher demands on the model’s ability to extract local features and ensure stable overall classification. As each patient is represented by a single independent image, this allows for a direct assessment of the classification performance of different models on independent patient-level test samples. The results of the model evaluation are shown in Table 3.

**Table 3:** Comparative classification performance on the PU2756 dataset.

Structure	Model	Accuracy	Macro precision	Macro recall	Macro F1-score
CNN-style	ResNet50	0.5822 ± 0.0371	0.5514 ± 0.0252	0.5493 ± 0.0265	0.5454 ± 0.0269
	EfficientNet-V2	0.5928 ± 0.0189	0.5726 ± 0.0174	0.5756 ± 0.0200	0.5707 ± 0.0191
	ConvNeXt-V2	0.5286 ± 0.0223	0.5065 ± 0.0158	0.5069 ± 0.0169	0.5029 ± 0.0164
	RepViT	0.5438 ± 0.0213	0.5270 ± 0.0211	0.5286 ± 0.0222	0.5246 ± 0.0213
Transformer-style	Vision-Transformer	0.5612 ± 0.0186	0.5375 ± 0.0149	0.5387 ± 0.0150	0.5363 ± 0.0144
	Swin-Transformer-V2	0.5420 ± 0.0059	0.5144 ± 0.0123	0.5151 ± 0.0128	0.5138 ± 0.0119
	EfficientViT	0.5500 ± 0.0370	0.5483 ± 0.0230	0.5515 ± 0.0245	0.5394 ± 0.0311
Hybrid-style	CoAtNet	0.5982 ± 0.0181	0.5774 ± 0.0108	0.5797 ± 0.0105	0.5752 ± 0.0101
	MaxViT	0.5891 ± 0.0139	0.5672 ± 0.0213	0.5704 ± 0.0235	0.5667 ± 0.0210
	MedViT	0.5906 ± 0.0354	0.5714 ± 0.0203	0.5720 ± 0.0217	0.5652 ± 0.0255
Mamba-style	EfficientViM	0.5692 ± 0.0248	0.5355 ± 0.0202	0.5350 ± 0.0193	0.5343 ± 0.0199
	MambaVision	0.5659 ± 0.0232	0.5473 ± 0.0103	0.5490 ± 0.0084	0.5444 ± 0.0120
	MedMamba	0.5688 ± 0.0299	0.5363 ± 0.0381	0.5379 ± 0.0375	0.5353 ± 0.0394
Proposed	Seed-Net	0.6054 ± 0.0086	0.5805 ± 0.0220	0.5828 ± 0.0267	0.5785 ± 0.0229

PU2756 produced substantially lower classification performance than TN5000 and SMC-LUD. Because malignant cases accounted for approximately 63% of the test set, an all-malignant majority-class baseline would achieve an Accuracy of approximately 0.63, but a Macro Recall of 0.50 and a Macro F1-score of approximately 0.39. Accuracy alone is therefore insufficient for interpreting performance on this dataset. Among the evaluated learning-based models, SEED-Net achieved the highest mean Macro Precision, Macro Recall, and Macro F1-score, reaching 0.5805 ± 0.0220, 0.5828 ± 0.0267, and 0.5785 ± 0.0229, respectively. Although its Accuracy of 0.6054 ± 0.0086 remained below the majority-class baseline, its higher class-balanced metrics indicate that it did not collapse to majority-class prediction. Nevertheless, its absolute performance remained limited. CoAtNet was the closest competing method, while SEED-Net’s advantage was modest, it remained consistent across different evaluation dimensions, indicating that its improvements were not driven by a single category or metric alone.

No consistent architectural advantage emerged among the other competing models, the Accuracy of EfficientNet-V2, MaxViT and MedViT was all close to 0.59, while their Macro F1-scores clustered between 0.56 and 0.57. Mamba-class models achieved Macro F1-scores of 0.5343–0.5444, failing to outperform convolutional or hybrid architectures. In the PU2756 task, the adoption of more complex attention or state-space structures did not directly translate into higher classification performance, local representation methods and class balance capabilities remain the primary factors influencing the results. Overall, SEED-Net achieved the highest mean Macro F1-score among the evaluated learning-based models under the current protocol, although its absolute performance remained limited.

### 4.4 Grad-CAM visualization analysis

**Figure 2:**
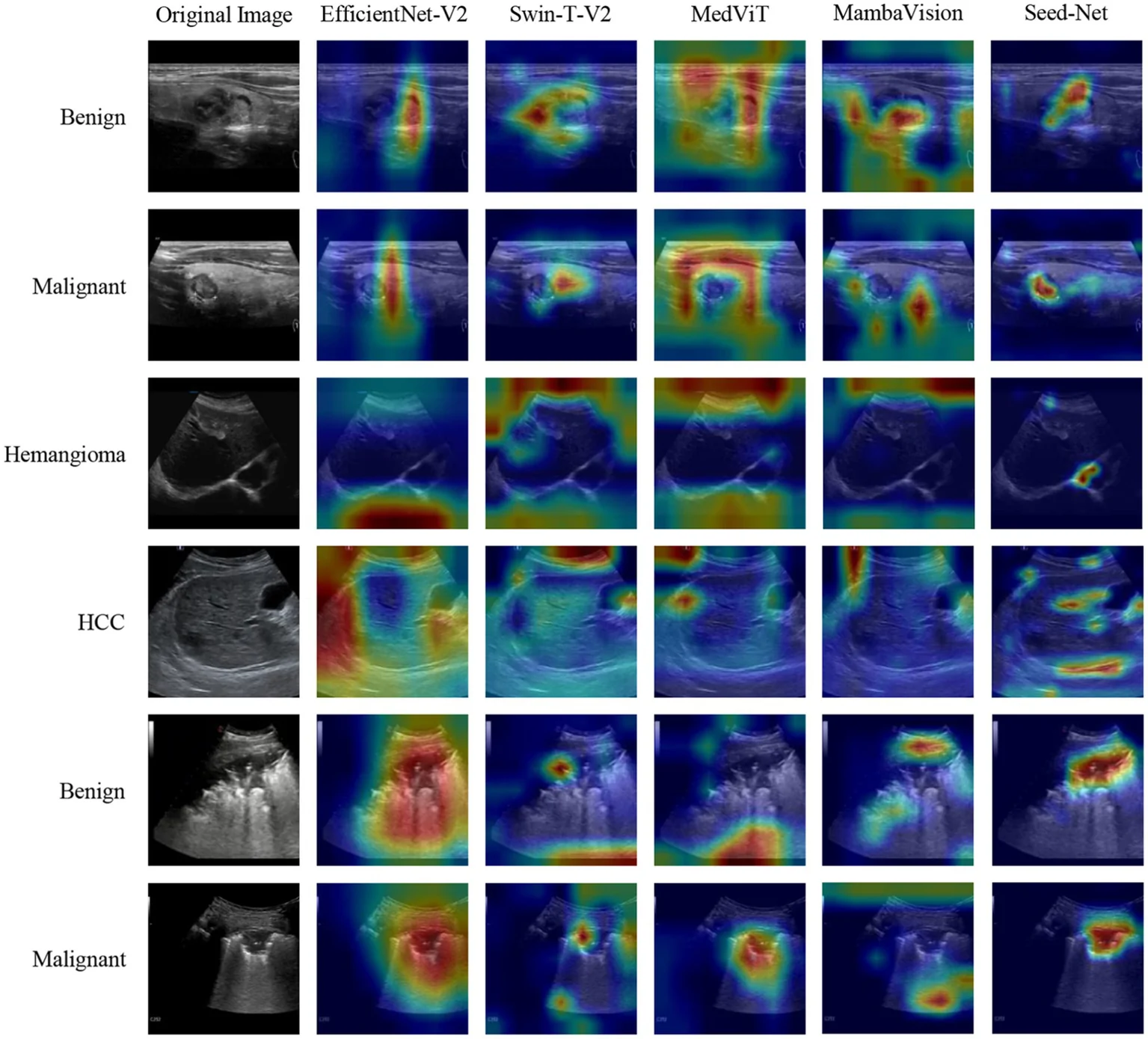
Grad-CAM visualization comparison of representative models on different datasets.

To further analyse the decision regions of different architectures, this study utilised Grad-CAM to visualise representative models. TN5000 serves as the primary experimental dataset in this paper. Based on evaluation metrics, the models with the highest Macro F1-scores, EfficientNet-V2, Swin-Transformer-V2, MedViT and MambaVision, were selected from the CNN, Transformer, Hybrid architecture and Mamba-class models respectively, and compared with SEED-Net, as shown in Figure 2. Standard Grad-CAM was used for all visualisations. For EfficientNet-V2, the target layer was the final convolutional feature block immediately before global average pooling. For Swin-Transformer-V2, the target layer was the first normalisation layer of the final Transformer block in the last stage. For MedViT, the output of the final MedViT block in the fourth stage, immediately before global feature aggregation, was selected. For MambaVision, the first normalisation layer of the final block in the last hierarchical stage was used. For SEED-Net, the output of the final SEED residual block in Stage 4, immediately before the Statistical Echo Evidence Head, was selected. For architectures producing token-form features, the activations were reshaped according to their original spatial grid before CAM calculation. All activation maps were generated with respect to the predicted-class logit, normalised to [0,1], and bilinearly resized to the input resolution for visualisation. These layers were selected because they represent the deepest architecture-specific features that retain spatial information before global aggregation and classification. To ensure consistency in model selection, the same model combinations were further applied to SMC-LUD and PU2756, without re-selecting models based on the results of each dataset. One test example from each class was selected from each dataset, resulting in six illustrative cases. The same images were used for all compared models. These examples were used only to qualitatively inspect activation patterns around the lesion, adjacent tissue, and image background, they were not used to quantify localisation performance or to rank the spatial accuracy of different architectures.

As can be seen from Figure 2, there are notable differences in the response to the target region across different architectures. In the benign and malignant samples of TN5000, the high-response region of EfficientNet-V2 extends vertically, while the activation ranges of MedViT and MambaVision are relatively dispersed and cover some adjacent tissue. Swin-Transformer-V2 is able to focus on the nodule region, but its response range remains relatively broad. In contrast, the high-response regions of SEED-Net are primarily concentrated near the nodule and its margins, with minimal activation in the background areas. On the SMC-LUD dataset, some of the comparison models exhibit strong responses to fan-shaped imaging boundaries, superficial regions or the lower edge of the image, this is particularly evident in haemangioma samples. SEED-Net’s responses are more concentrated on the local lesion and its adjacent hepatic parenchyma. In HCC samples, all models exhibited some degree of multi-region activation, SEED-Net primarily retained local responses within and around the lesion. In PU2756, EfficientNet-V2’s response covered the lesion and a large area of surrounding tissue, while other comparison models were affected to varying degrees by superficial regions, image edges or posterior acoustic regions. SEED-Net exhibits a more concentrated local response in both benign and malignant samples, primarily covering the main body of the lesion and its peripheral regions. Overall, in the displayed examples, SEED-Net exhibited a more compact activation range and fewer responses in non-lesion regions. These observations provide qualitative support that lesion-related and adjacent-tissue features contributed to the predictions. However, Grad-CAM does not provide causal evidence and cannot conclusively exclude residual shortcut learning from image layout, acquisition conditions, or background tissues. Because the heatmaps were generated from architecture-specific feature layers, their spatial extent should not be interpreted as a directly comparable quantitative measure across models.

### 4.5 Class-wise prediction analysis

**Figure 3:**
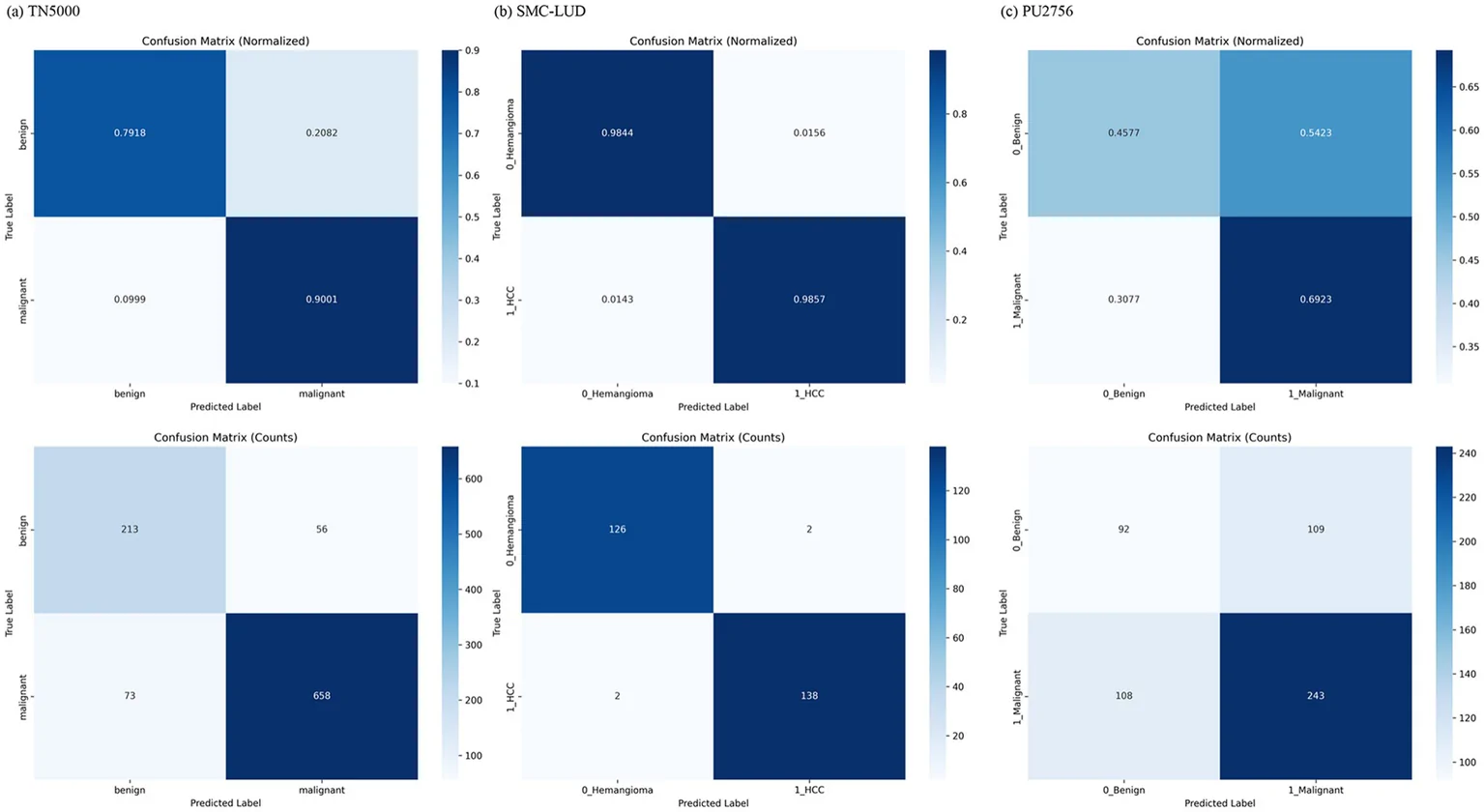
Confusion matrices of SEED-Net on the **(a)** TN5000, **(b)** SMC-LUD, and **(c)** PU2756 test sets.

To further analyse the class-level performance of SEED-Net across different ultrasound classification tasks, Figure 3 presents both row-normalised and count-based confusion matrices for the TN5000, SMC-LUD and PU2756 test sets. For each dataset, the matrices were generated from a representative single run among the five random-seed experiments. The selected run showed overall test performance closest to the corresponding five-run mean and was used consistently for both the Grad-CAM visualisation and the confusion-matrix analysis. In Figure 3, rows represent the true classes and columns represent the predicted classes. The upper row reports proportions normalised by the corresponding true class, whereas the lower row reports the absolute sample counts obtained from the same test predictions.

SEED-Net exhibits different class-level error patterns across the three tasks. With malignant nodules treated as the positive class, the TN5000 confusion matrix showed a sensitivity of 90.01% and a specificity of 79.18%. The primary source of error stems from benign nodules being misclassified as malignant, indicating that, when using whole-image input, the borders and internal echo patterns of some benign nodules can easily be confused with malignant features. In SMC-LUD, the recognition rates for haemangioma and HCC were 98.44 and 98.57% respectively, on clean images with caliper markers removed, the two types of lesions could be distinguished with relative stability. For PU2756, the benign-class recall, corresponding to specificity, was 45.77%, whereas the malignant-class recall, corresponding to sensitivity, was 69.23%. The substantially lower specificity indicates that benign lesions were frequently classified as malignant, reflecting the considerable overlap between benign and malignant pulmonary tumour appearances under the current single-frame B-mode setting. Overall, SEED-Net achieved the clearest class separation on SMC-LUD and showed stronger recognition of malignant nodules on TN5000. PU2756 represented the most challenging evaluation setting and highlighted the limitations of single-frame B-mode ultrasound for reliable benign–malignant pulmonary tumour classification under the current protocol. As a potential image-analysis assistance framework, SEED-Net could provide supplementary image-level evidence for radiologists when reviewing selected static B-mode images. For example, it may support the reassessment of cases in which image appearances are ambiguous, while the class-specific results and confusion patterns can indicate situations requiring greater diagnostic caution. However, the model should not be interpreted as an independent diagnostic system, and its effect on radiologist performance and clinical decision-making remains to be established through prospective reader studies and independent clinical validation.

### 4.6 Ablation experiment

To evaluate the contributions of individual components under different organ-specific ultrasound settings, the ablation study was first conducted on TN5000, the largest dataset and the primary experimental dataset in this study, and was subsequently replicated on PU2756, the most challenging dataset in terms of absolute classification performance. For each dataset, all ablation variants adopted the same data partition and training strategy as the corresponding complete model. The ablation experiments consisted of two parts: progressive construction and single-component removal. The progressive construction experiment first utilised the Residual Baseline, retaining only standard residual backbone with the same depth and channel configuration as the full model, and employing GAP and a Linear Classifier. Subsequently, its classification head was replaced with the Statistical Echo Evidence Head (SEEH) to form Residual + SEEH, which was used to evaluate the overall effect of statistical pooling, mid-layer evidence fusion and normalised classification decisions. Finally, the complete SEED-Net backbone was introduced to obtain the full model (Table 4).

**Table 4:** Ablation results and computational complexity of SEED-Net on TN5000.

Variant	Accuracy	Macro precision	Macro recall	Macro F1-score	Params	FLOPs
Residual	0.8598 ± 0.0104	0.8215 ± 0.0141	0.8245 ± 0.0111	0.8227 ± 0.0117	11.1875	1.7733
Residual + SEEH	0.8638 ± 0.0133	0.8279 ± 0.0204	0.8324 ± 0.0123	0.8287 ± 0.0121	12.1050	1.7742
w/o FTG	0.8596 ± 0.0059	0.8182 ± 0.0070	0.8394 ± 0.0094	0.8274 ± 0.0076	13.0145	1.9636
w/o GDU	0.8564 ± 0.0117	0.8179 ± 0.0170	0.8249 ± 0.0090	0.8201 ± 0.0093	12.1050	1.7742
w/o CR	0.8664 ± 0.0048	0.8317 ± 0.0048	0.8271 ± 0.0162	0.8289 ± 0.0093	13.0144	1.9636
w/o MEEF	0.8658 ± 0.0103	0.8289 ± 0.0150	0.8370 ± 0.0098	0.8319 ± 0.0092	12.6207	1.9632
w/o MSSD	0.8630 ± 0.0072	0.8231 ± 0.0083	0.8384 ± 0.0156	0.8299 ± 0.0108	12.2275	1.9628
w/o CNC	0.8630 ± 0.0088	0.8265 ± 0.0080	0.8292 ± 0.0292	0.8263 ± 0.0174	13.0144	1.9636
SEED-Net	0.8702 ± 0.0055	0.8324 ± 0.0081	0.8459 ± 0.0037	0.8384 ± 0.0048	13.0145	1.9636

The single-module removal experiments are based on the complete SEED-Net, with separate configurations of w/o Foreground Tissue Gate, w/o Guided Detail Unit and w/o Channel Recalibration, to analyse the independent contributions of foreground tissue modulation, residual modelling of local structures and details, and channel recalibration. For the Statistical Echo Evidence Head, further variations were set up w/o Mean-Std Statistical Descriptors and w/o Mid-level Echo Evidence Fusion, while the Cosine-normalised Classifier was replaced with a standard Linear Classifier, thereby evaluating the impact of statistical descriptors, multi-level evidence fusion and the form of classification decision-making on the final performance. Apart from the target module, the remaining architecture and training settings for each ablation variant remained unchanged. All results are reported as the mean and standard deviation of five independent runs and are interpreted as descriptive comparisons rather than claims of statistical significance.

Stepwise evaluation results show that Residual + SEEH improves Accuracy and Macro F1-score by 0.4 and 0.6 percentage points, respectively, compared to the Residual Baseline. Upon further introduction of the full SEED-Net backbone, both metrics continue to improve to 0.8702 and 0.8384. Compared with the baseline model, the full model’s Macro Recall increased by 2.14 percentage points, a greater increase than that of Accuracy, indicating that the overall improvement is primarily reflected in the model’s balanced recognition capability across both sample categories. The number of model parameters increased from 11.19 M to 13.01 M, while FLOPs rose from 1.77G to 1.96G, representing a relatively limited increase in computational overhead.

**Figure 4:**
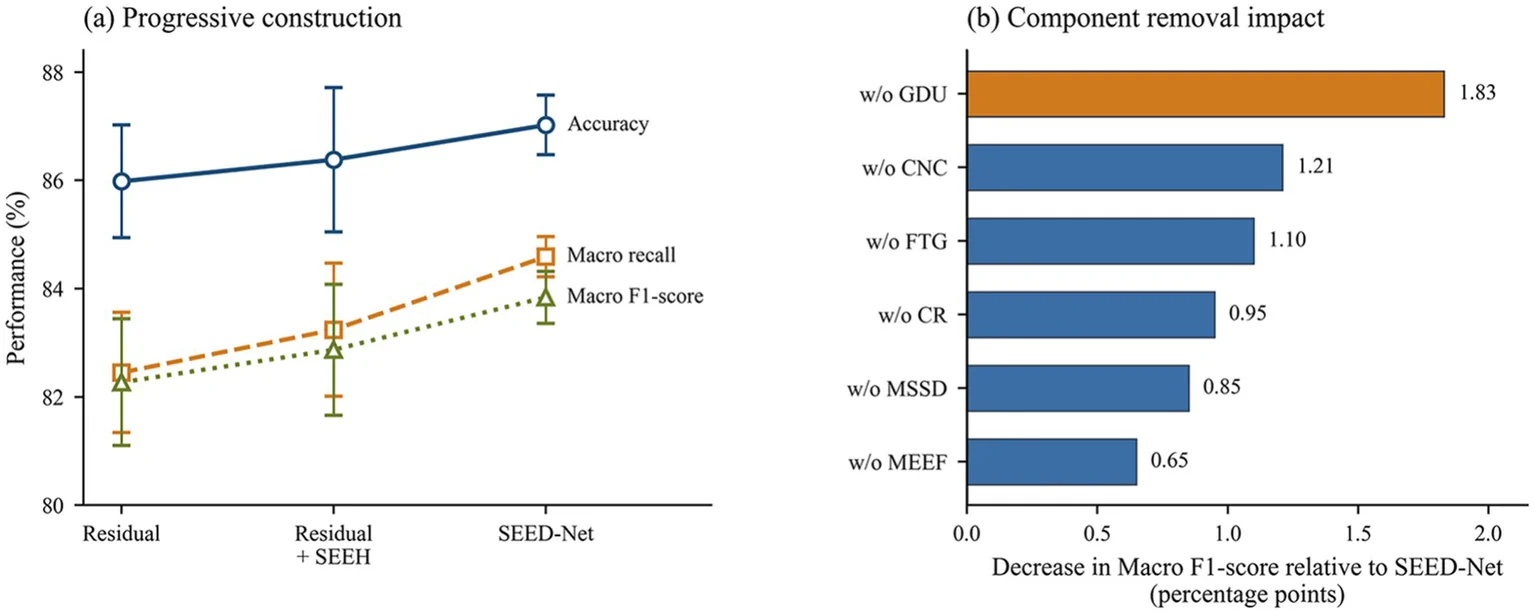
**(a)** Progressive construction and **(b)** component removal impact ablation analysis of SEED-Net.

Upon removal of individual modules, none of the four metrics of the complete model were surpassed. On TN5000, removal of the Guided Detail Unit produced the largest observed mean reduction in Macro F1-score among the evaluated variants, as shown in Figure 4, decreasing it by 1.83 percentage points. Accuracy and Macro Recall decreased by 1.38 and 2.10 percentage points, respectively. Replacing the cosine-normalised classifier with a linear classifier resulted in a 1.21 percentage decrease in the Macro F1-score, and the fluctuations in Macro Recall increased significantly. Within the complete SEED-Net configuration, removing FTG or CR reduced the mean Macro F1-score by 1.10 and 0.95 percentage points, respectively. These reductions represent their conditional contributions in the presence of GDU and the remaining components, rather than independent additive effects. Removal of CR also reduced Macro Recall by 1.88 percentage points, indicating that its conditional contribution on TN5000 was mainly reflected in class-balanced recognition. Removing the Mean–Std Statistical Descriptors and Mid-level Echo Evidence Fusion resulted in decreases in the Macro F1-score of 0.85 and 0.65 percentage respectively, indicating that both provide stable but relatively modest gains. The component-removal results should be interpreted as conditional rather than independently additive effects. Residual + SEEH contains none of FTG, GDU, or CR, w/o FTG retains GDU and CR, w/o GDU retains FTG and CR, w/o CR retains FTG and GDU, and the complete SEED-Net contains all three components. Their Macro F1-scores on TN5000 were 0.8287, 0.8274, 0.8201, 0.8289, and 0.8384, respectively. The lower result of w/o GDU compared with Residual + SEEH indicates that FTG and CR did not provide an independent improvement in the absence of GDU. In contrast, the complete three-component configuration achieved the highest mean Macro F1-score, suggesting that FTG and CR operate conditionally on the structure–detail representation established by GDU. Overall, the progressive-construction and component-removal results on TN5000 support the complementary roles of the proposed components, with removal of GDU producing the largest observed mean reduction in Macro F1-score.

On PU2756, progressive construction increased the Macro F1-score from 0.5584 for the residual baseline to 0.5751 after introducing SEEH and to 0.5785 for the complete SEED-Net. This result indicates that both the statistical evidence head and the SEED backbone contributed to the performance improvement under the pulmonary ultrasound setting.

Among the component-removal variants, removal of GDU produced the largest observed mean decrease in Macro F1-score, reducing it to 0.5710, which was 0.75 percentage points lower than the complete model. Removal of CNC and FTG also resulted in lower mean Macro F1-scores of 0.5767 and 0.5777, respectively. In contrast, removal of CR, MEEF, or MSSD resulted in only marginal fluctuations around the complete model, and these differences were small relative to the variability across repeated runs. These results indicate that the contribution of the smaller auxiliary components was more dataset-dependent on PU2756, whereas GDU showed the most consistent reduction when removed on both TN5000 and PU2756 (Table 5).

**Table 5:** Ablation results of SEED-Net on the PU2756 dataset.

Variant	Accuracy	Macro precision	Macro recall	Macro F1-score
Residual	0.5833 ± 0.0126	0.5593 ± 0.0093	0.5612 ± 0.0104	0.5584 ± 0.0096
Residual + SEEH	0.6080 ± 0.0173	0.5781 ± 0.0175	0.5774 ± 0.0197	0.5751 ± 0.0190
w/o FTG	0.6058 ± 0.0141	0.5778 ± 0.0123	0.5788 ± 0.0120	0.5777 ± 0.0124
w/o GDU	0.5971 ± 0.0347	0.5733 ± 0.0237	0.5737 ± 0.0216	0.5710 ± 0.0248
w/o CR	0.5971 ± 0.0215	0.5839 ± 0.0177	0.5875 ± 0.0183	0.5793 ± 0.0149
w/o MEEF	0.6058 ± 0.0192	0.5797 ± 0.0226	0.5818 ± 0.0243	0.5798 ± 0.0231
w/o MSSD	0.6109 ± 0.0196	0.5836 ± 0.0153	0.5824 ± 0.0159	0.5797 ± 0.0144
w/o CNC	0.5884 ± 0.0255	0.5817 ± 0.0208	0.5875 ± 0.0223	0.5767 ± 0.0231
SEED-Net	0.6054 ± 0.0086	0.5805 ± 0.0220	0.5828 ± 0.0267	0.5785 ± 0.0229

The pairwise configurations further showed that w/o GDU, which retained FTG and CR, achieved a Macro F1-score of 0.5710, lower than the 0.5751 obtained by Residual + SEEH. This pattern is consistent with the TN5000 result and indicates that FTG and CR do not provide a stable independent gain without GDU. In contrast, the small fluctuations observed after removing CR or the internal SEEH components indicate that their effects were more dataset-dependent on PU2756.

## 5 Conclusion

This paper proposed SEED-Net for the classification of two-dimensional B-mode ultrasound images. Through the Foreground Tissue Gate, Guided Detail Unit, Channel Recalibration and Statistical Echo Evidence Head, it jointly models the response of foreground tissue, local structural differences, statistical information from medium to deep layers, and class-discriminative features. Each prior branch is initialised using near-zero initialisation, enabling the model to maintain a stable residual skeleton during the early stages of training, before gradually learning features modulated by ultrasound echoes. Experiments on the TN5000, SMC-LUD and PU2756 datasets demonstrate that SEED-Net maintains strong overall performance across three independently evaluated organ-specific ultrasound classification tasks with varying levels of difficulty, while keeping the number of parameters and computational complexity low. Progressive-construction and component-removal experiments on TN5000 and PU2756 indicated complementary roles for backbone detail modelling and the statistical evidence head. Removal of GDU produced the largest observed mean reduction in Macro F1-score on both ablation datasets, whereas the contributions of the remaining auxiliary components varied with the dataset. Grad-CAM and confusion matrices further demonstrate that the model is able to focus on lesions and adjacent tissues in the majority of samples, although notable category confusion still persists in the PU2756 dataset. Although obvious device-interface regions were removed and Grad-CAM responses were generally concentrated on lesions and adjacent tissues, the current experiments cannot completely exclude acquisition-, layout-, or background-related shortcut features within individual datasets. The present results characterise image-level classification of preselected static B-mode frames rather than a complete clinical ultrasound examination. Further research is required to validate the stability and generalisation of SEED-Net on multi-centre external datasets and to extend the current evaluation to case-level, multi-frame and clinically richer ultrasound classification settings.

## Data Availability

The original contributions presented in the study are included in the article/supplementary material, further inquiries can be directed to the corresponding author.
